# Health-Related Quality of Life in Patients With ANCA-Associated Vasculitis and Sinonasal Involvement: A Single-Center Cross-Sectional Study

**DOI:** 10.1097/RHU.0000000000001630

**Published:** 2020-11-02

**Authors:** Diego Cazzador, Roberto Padoan, Roberta Colangeli, Alfonso Luca Pendolino, Mara Felicetti, Elisabetta Zanoletti, Enzo Emanuelli, Alessandro Martini, Andrea Doria, Piero Nicolai, Franco Schiavon

**Affiliations:** From the ∗ Otorhinolaryngology Unit; † Section of Human Anatomy, Department of Neurosciences; ‡ Rheumatology Unit, Department of Medicine DIMED, University of Padua, Padua, Italy.

**Keywords:** ANCA-associated vasculitis, granulomatosis with polyangiitis, microscopic polyangiitis, quality of life, SNOT-22

## Abstract

**Background/Objective:**

The aim of this study was to assess the impact of sinonasal morbidity on quality of life (QoL) in antineutrophil cytoplasmic antibody–associated vasculitis (AAV).

**Methods:**

This cross-sectional case-control study enrolled 71 patients—44 AAV cases with (ear, nose, and throat [ENT]–AAV) or without ENT involvement (non–ENT-AAV) undergoing multidisciplinary evaluations and 27 chronic rhinosinusitis (CRS) cases. Three validated QoL questionnaires (Sino-Nasal Outcomes Test-22 [SNOT-22], Nasal Obstruction Symptom Evaluation [NOSE], and Short-Form 36) were administered, and the 3 groups were compared.

**Results:**

The ENT-AAV patients were significantly younger (*p* = 0.01), with less antineutrophil cytoplasmic antibody positivity frequency (*p* = 0.035) and lower renal involvement (*p* = 0.003) than the non–ENT-AAV patients.

The SNOT-22 questionnaire demonstrated significantly greater sinonasal morbidity in ENT-AAV patients compared with CRS patients (*p* < 0.001). The NOSE score of ENT-AAV patients was comparable to those of CRS patients, but higher than that of non–ENT-AAV patients (*p* < 0.001). The SNOT-22 and NOSE scores positively correlated with disease activity (*p* = 0.037; *p* = 0.004, respectively). Short-Form 36 domain-by-domain analysis revealed a significantly poorer QoL in ENT-AAV patients, especially with physical functioning being progressively impaired in CRS, non–ENT-AAV, and ENT-AAV patients (*p* < 0.001). No significant differences in QoL came to light when AAV patients were stratified according to current systemic o local treatments.

**Conclusions:**

The QoL in AAV patients is significantly reduced, especially in the presence of ENT involvement. The AAV-related nasal morbidity is consistent and comparable to that reported by CRS patients. It significantly affects patients' QoL and in particular social functioning, leading to limitation in daily/work activities. Organ-focused questionnaires and multidisciplinary management are warranted to pursue a treat-to-target approach in these patients.

Granulomatosis with polyangiitis (GPA), formerly known as Wegener granulomatosis, and microscopic polyangiitis (MPA) are rare systemic diseases characterized by autoimmune necrotizing vasculitis of small and medium vessels that are classified among the antineutrophil cytoplasmic antibody (ANCA)–associated vasculitides (AAVs).^1^

Antineutrophil cytoplasmic antibody–associated vasculitides present with systemic involvement, classically affecting the upper and lower respiratory tract,^2^ kidneys, joints, and nerves^3^ accompanied by fever, weight loss, and fatigue.^4^ Granulomatosis with polyangiitis and MPA share clinical and histopathological features, a tight link with positive ANCA serology, and similar treatment modalities.^5,6^ Therefore, they may represent a continuum of disease phenotypes with a predominantly granulomatous or vasculitic disease pattern; risk of relapse is linked to the granulomatous component, whereas mortality is associated with vasculitis.^7^ Newer treatment regimens have modified the clinical course of AVV, from an acute form, characterized by high mortality rates, to a chronic condition.^8,9^ Despite treatment, the risk of relapse is 30% to 50% over 5 years.^10,11^ Many patients experience persistent disease activity, long-term exposure to toxic therapies, and almost one third of patients presents irreversible damage at diagnosis.^12^

Impaired health-related quality of life (QoL) has been reported in patients with AAV, especially as a consequence of fatigue and pain.^4,13–19^ Moreover, it is reported that one fourth of patients experience depression and more than 40% present anxiety.^17^ Work disability is also high with approximately 25% unemployed^20^ and 50% reporting that their career is hampered because of AAV.^18^

Ear, nose, and throat (ENT) involvement in AAV, especially GPA, represents one of the most frequent symptoms.^21^ Although patients with ENT symptoms have better survival^2,22^ and less renal involvement,^2,23^ they typically present a relapsing disease.^24,25^ The consequently impaired health-related QoL caused by ENT symptoms is considered to be even worse than other severe conditions, such as dialysis, seizures, and oxygen dependence.^13,26,27^

The aim of the present study is to evaluate the sinonasal morbidity and its impact on global QoL in patients with AVV.

## PATIENTS AND METHODS

The present study was carried out at the University of Padua in accordance with the National Health Research Ethics guidelines, and thus formal ethical approval was not required. However, informed consent was obtained from each subject before starting any study-related procedure.

A cross-sectional case-control study was performed through collaboration between the Otorhinolaryngology and Rheumatology Units. We enrolled patients with an established diagnosis of GPA or MPA with a minimum disease duration of 6 months, regularly followed at the Rheumatology Unit, and patients with chronic rhinosinusitis (CRS) matched by age and sex, followed at the Otorhinolaryngology Unit. Patients with AAV at disease onset or with relapsing disease were excluded to avoid possible confoundment.

Granulomatosis with polyangiitis or MPA and CRS were classified according to the European Medicines Agency algorithm^28,29^ (including biopsies when deemed necessary) and the European Position Paper on Rhinosinusitis and Nasal Polyps guidelines,^30^ respectively.

All AAV patients were clinically evaluated by 3 expert rheumatologists (R.P., M.F., and F.S.) and assessed for disease activity and damage, using the Birmingham Vasculitis Activity Score (BVAS) version 3^31^ and the Vasculitis Damage Index (VDI),^32^ respectively. Data on demographics and clinical features, as well as organ involvement and laboratory findings, were collected. Patients' treatment details including immunosuppressive agents, glucocorticoids, topical nasal treatments, and history of nasal surgery were also noted. The ENT evaluation consisted in nasal endoscopic examination with high-definition rigid endoscopes to ascertain the presence of nasal inflammation, nasal crusting, nasal discharge, septal perforation, or bony erosion. Three validated questionnaires were administered to each patient. To widen the spectrum of sinonasal symptoms assessment, the Sino-Nasal Outcomes Test-22 (SNOT-22)^33^ and Nasal Obstruction Symptom Evaluation (NOSE)^34^ were applied. Sino-Nasal Outcomes Test-22 results were analyzed as total score and domains score.^35^ Nasal Obstruction Symptom Evaluation total scores were multiplied by 5 to reach a score of 0 to 100. A modified sinonasal outcome test score (SNOT-25), which included 3 additional sinonasal symptoms (nasal crusting, bleeding, and external nasal deformity) specific for AAV patients, was also calculated.^26^ Moreover, the Medical Outcome Study Short-Form 36 (SF-36),^36^ which is commonly used for the measurement of QoL and consists of 8 domains investigating physical function, physical role, bodily pain, general health, vitality, social functioning, emotional role, and mental health, was also administered. Responses were scored for their perceived current disease activity.

Because the questionnaires investigate not only the sinonasal manifestations but also extranasal and psychological involvement, they were administered to all the patients, irrespective of ENT involvement. For further analysis, AAV patients were divided into 2 groups (ENT-AAV and non–ENT-AAV) depending on the presence of active sinonasal involvement at the time of the survey. The non–ENT-AAV group and the CRS group served as double controls to the ENT-AAV group.

### Statistical Analysis

All data were entered into a computerized anonymized database. Statistical analysis was carried out with SPSS 24.0 and GraphPad Prism 7.0 software. The hypothesis of normality was tested with d'Agostino-Pearson test. Categorical variables were expressed with absolute frequencies and percentages, continuous variables as mean average and standard deviation, and median and interquartile range (IQR) or median with maximum and minimum based on the distribution of the variable. Dichotomous or ordinal categorical variables were compared with the χ^2^ test or the Fisher exact test. Continuous variables were studied with *t* tests for dependent and independent samples or with the nonparametric Mann-Whitney *U* test for independent samples based on data distribution. Spearman correlation was also used to identify associations between continuous variables. The level of significance was α = 0.05.

## RESULTS

The study included 71 patients—44 (62.0%) with AAV and 27 (38.0%) with CRS. Within the AAV group, 20 GPA patients (45.5%) presented ENT involvement, whereas 24 patients (54.5%) without ENT symptoms were classified as GPA (n = 17, 70.8%) and MPA (n = 7, 29.2%).

### Population Demographics and Characteristics of Disease and Treatment

Clinical and demographic data on AVV patients at evaluation are summarized in Table 1. Comparing non–ENT-AAV and ENT-AAV patients, the former were older (63.5 years [52.2–71.0 years] vs 53 years [26.7–61.0 years], *p* = 0.021). Patients with ENT involvement presented a slightly higher disease activity index (BVAS 0 [0–3.7] vs 0 [0–0], *p* = 0.037) and a higher VDI for the ENT region (90.0% vs 30.0%, *p* < 0.001). No difference was noted in the inflammation indexes and total VDI score. At diagnosis, patients with ENT involvement were significantly younger (44 years [31–54 years] vs 56 years [46–56 years], *p* = 0.01), with less ANCA positivity frequency (80.0% vs 100%, *p* = 0.035) and lower renal involvement (20.0% vs 66.7%, *p* = 0.003), and with a history of acute kidney injury, rapidly progressive glomerulonephritis, or subacute/chronic nephritis. No significant differences were observed between patients with ENT involvement and those without in terms of systemic treatment regimens (oral glucocorticoids and immunosuppressive agents) and disease duration. Additional details on patients' characteristics are reported in Table 1.

**TABLE 1:** Demographic, Clinical, and Laboratory Characteristics of Patients With ENT-AAV, Non–ENT-AAV, and CRS

	ENT-AAV (n = 20)	Non–ENT-AAV (n = 24)	*p* value	CRS (n = 27)	*p* value
Age, y	53.0 (26.7–61.0)	63.5 (52.2–71.0)	**0.0214**	56.0 (38.0–68.0)	NS
Female, n (%)	11 (55.0)	13 (54.2)	NS	11 (40.7)	NS
Disease duration (IQR), mo	49.5 (6–196)	68.0 (6–248)	NS	—	—
Organ involvement, n (%)				—	—
Systemic	12 (60.0)	17 (70.8)	NS		
Lung	11 (55.0)	15 (62.5)	NS		
Skin	4 (20.0)	8 (33.3)	NS		
Eye	3 (15.0)	3 (12.5)	NS		
Cardiovascular	0 (0)	1 (4.2)	NS		
Abdominal	4 (20.0)	2 (8.3)	NS		
Renal	4 (20.0)	16 (66.7)	**0.0027**		
Nervous	6 (30.0)	12 (50.0)	NS		
ANCA positivity, n (%)	16 (80.0)	24 (100)		—	—
ANCA PR3	15 (93.7)	13 (54.2)	**0.0357**		
ANCA MPO	1 (6.3)	11 (45.8)			
BVAS v3	0 (0–10)	0 (0–7)	**0.0374**	—	—
BVAS v3 > 0, n (%)	7 (35.0)	2 (8.3)	**0.028**	—	—
VDI	3 (2–4)	3 (2–4)	NS	—	—
ESR, mm/h	21 (9–35)	22.5 (9–55)	NS	—	—
CRP, mg/L	1.8 (0.3–7.8)	1.9 (0.5–24.5)	NS	—	—
Immunosuppressive agents, n (%)	11 (55.0)	13 (54.2)	NS	—	—
DMARDs, n (%)	11 (100)	12 (92.3)	NS		
Rituximab, n (%)	0 (0)	1 (7.7)	NS		
Oral glucocorticoids, n (%)	10 (50.0)	13 (54.2)	NS	—	—
Oral glucocorticoids, mg/d	1.25 (0–7.5)	3.25 (0–5)	NS		
Oral antibiotic (cotrimoxazole), n (%)	2 (10.0)	0 (0)	NS	—	—
Nasal steroid, n (%)	6 (30.0)	1 (4.2)	**0.0353**	16 (59.2)	**0.047**
Local antibiotic, n (%)	4 (20.0)	—	—	—	—
Nasal douche, n (%)	11 (55.0)	—	—	4 (14.8)	**0.0035**
Moisturizing ointments, n (%)	6 (30.0)	1 (4.2)	**0.0353**	3 (11.1)	NS

Values in bold mean that they are statistically significant (*p* < 0.05).

All data are presented as median and IQR, if not otherwise specified.

BVAS v3, Birmingham Vasculitis Activity Score version 3; ESR, erythrocytes sedimentation rate; CRP, C-reactive protein; DMARDs, disease-modifying antirheumatic drugs; MPO, myeloperoxidase; NS, nonsignificant; PR3, proteinase 3.

### Score Analysis

#### SNOT-22 Score

The SNOT-22 score was significantly different in the 3 groups of patients. Patients with AAV-ENT had a significantly higher score compared with non–ENT-AAV patients (33.5 [24.0–60.3] vs 14.5 [4.0–26.8], *p* < 0.001) and CRS patients (26.0 [17.0–38.0], *p* < 0.001). Among all ENT-AAV patients, 20.0% reported decreased sense of smell/taste, 15.0% ear fullness, 15.0% fatigue, 15.0% frustrated/restless/irritable, and 20.0% sad as 1 of their 5 most troublesome symptoms. The SNOT-22 symptoms domain analysis revealed significantly higher scores for the rhinologic, extranasal rhinologic, ear/facial, and psychological dysfunction domains in the ENT-AAV cohort, whereas no differences were observed for the sleep dysfunction domain. Similar results were also seen with the SNOT-25 questionnaire, where patients with ENT-AAV scored higher than patients with non–ENT-AAV (43.5 [27.8–63.8] vs 14.5 [4.0–26.8], *p* < 0.001) and CRS patients (28.0 [18.0–42.0], *p* < 0.001).

#### NOSE Score

**FIGURE 1:**
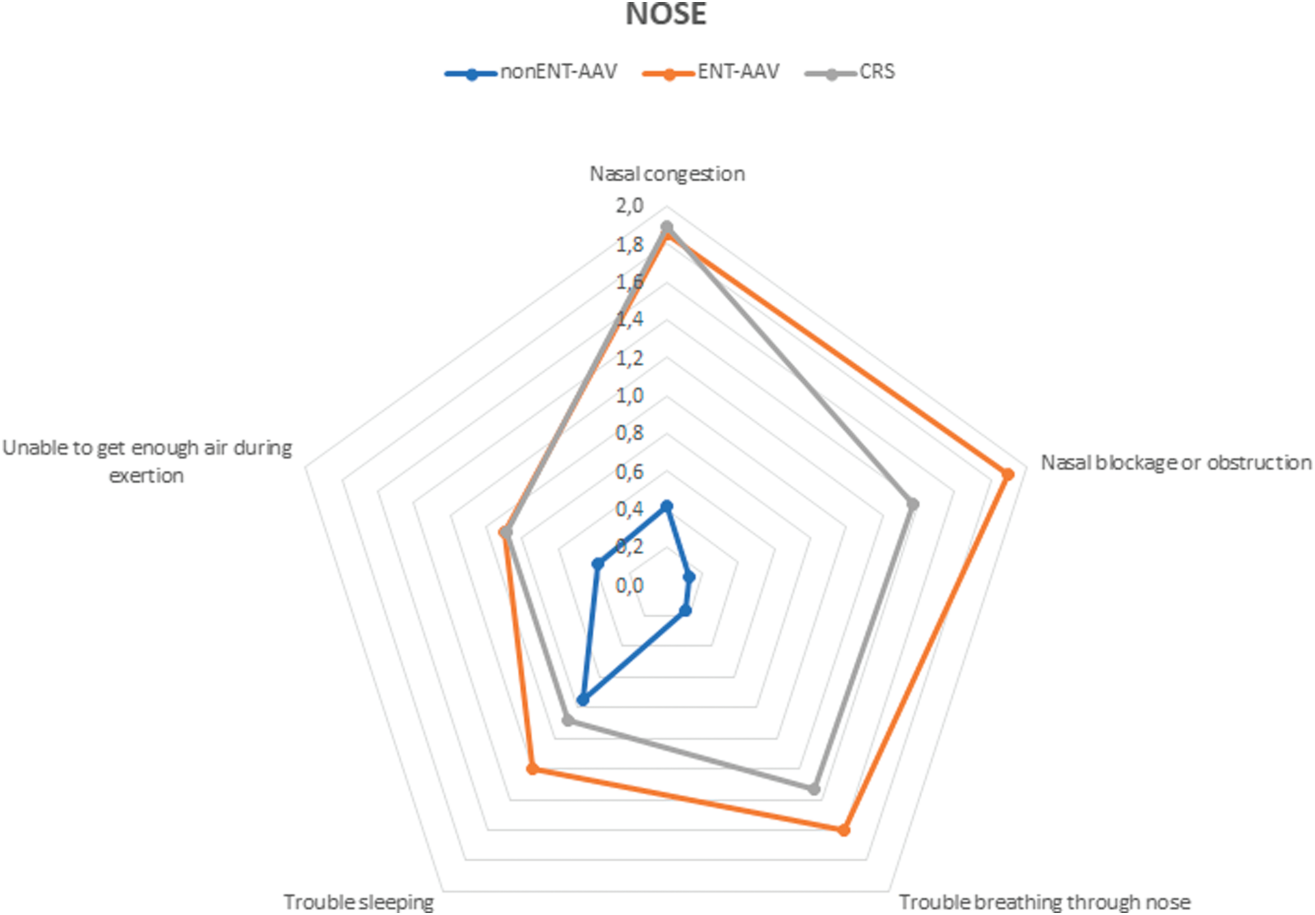
Radar chart displaying NOSE items comparison between the 3 groups of patients: non–ENT-AAV, ENT-AAV, and CRS.

The NOSE score was significantly different between ENT-AAV and non–ENT-AAV patients (30 [25–51] vs 5 [0–15], *p* < 0.001). Similar results were obtained comparing patients with CRS to those with non–ENT-AAV 30 [10–45] vs 5 [0–15], *p* < 0.001). The comparison between ENT-AAV and CRS was not significant in terms of medians and total NOSE scores. Considering comparison of single items between the 3 groups (Fig. 1), ENT-AAV scores were comparable to those of the CRS cohort, but significantly higher than non–ENT-AAV patients, whereas trouble in sleeping showed no difference. In addition, among all ENT-AAV patients, sleep dysfunction was rated as the least troublesome symptom (5.0%).

#### SF-36 Score

**FIGURE 2:**
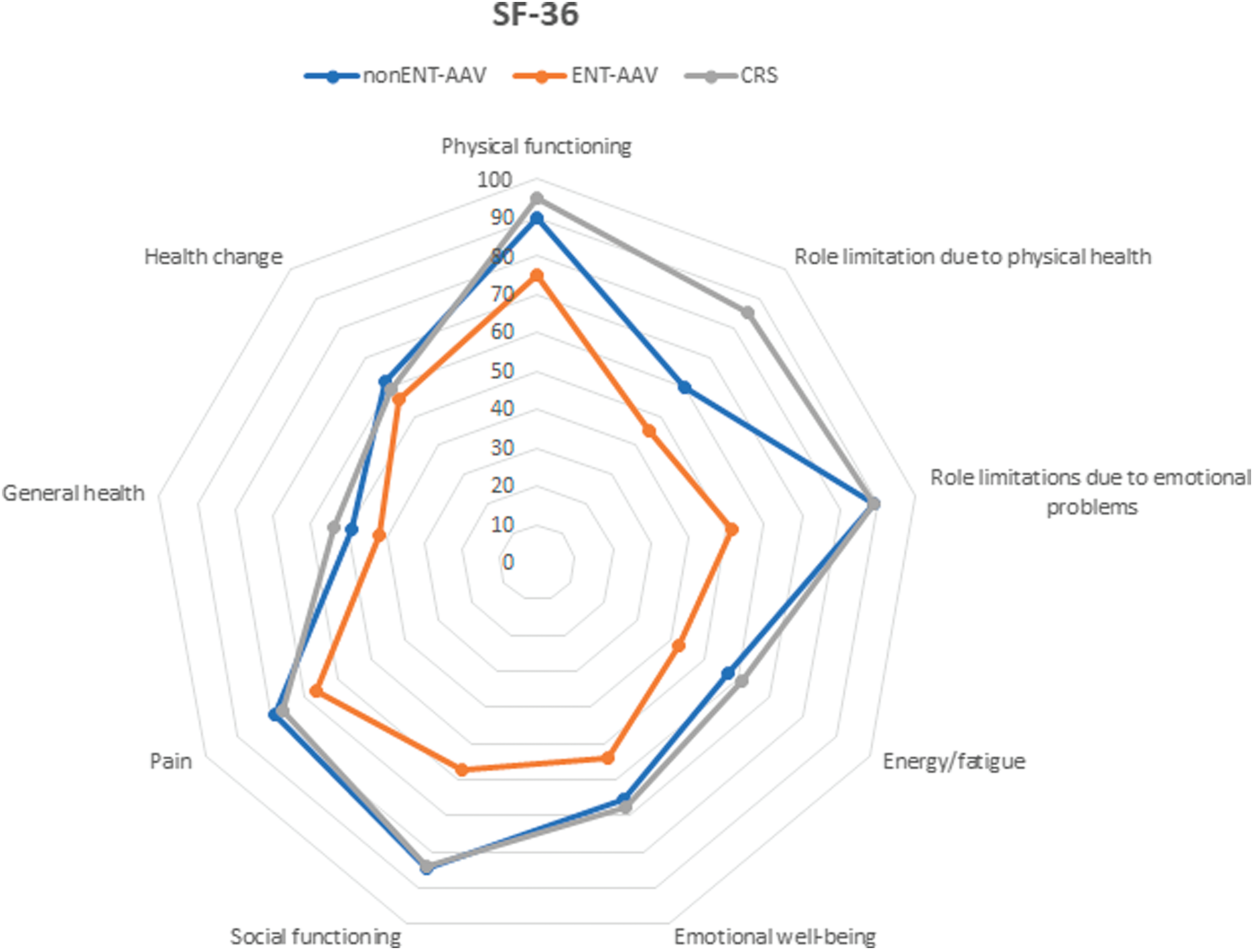
Radar chart displaying SF-36 domains comparison between the 3 groups of patients: non–ENT-AAV, ENT-AAV, and CRS.

Considering the SF-36 score (Fig. 2), physical functioning domains were significantly different among the 3 groups of patients, being progressively impaired in CRS (95.0%), non–ENT-AAV (90.0%), and ENT-AAV (75.0%) patients (Table 2). Antineutrophil cytoplasmic antibody–associated vasculitis patients demonstrated significant impairment of the role limitation because of physical health item in comparison to CRS patients (85.0%, 60.0%, and 45.0%). Furthermore, comparing ENT-AAV patients with non–ENT-AAV and CRS cohorts, significant impairment was observed in the role limitation because of emotional problems, energy/fatigue, and social functioning, whereas a statistical trend emerged for emotional well-being.

**TABLE 2:** Statistical Analysis of SF-36 Domain Scores in the 3 Cohorts of Patients

SF-36 Domains	CRS (n = 27)	*p* value	ENT-AAV (n = 20)	*p* value	non–ENT-AAV (n = 24)
Physical functioning	95.0 (95.0–100)	**<0.0001**	75.0 (66.3–90.0)	**0.0017**	90.0 (90.0–95.0)
Role limitations because of physical health	100 (75.0–100)	**0.0009**	50.0 (0–93.8)	**0.0257**	75.0 (6.3–100)
Role limitations because of emotional problems	100 (100–100)	**0.0012**	50.0 (0–100)	**0.0019**	100 (100–100)
Energy/fatigue	65.0 (50–70)	**0.005**	42.5 (20.0–67.5)	**0.0276**	60.0 (50.0–68.7)
Emotional well-being	68.0 (56.0–80.0)	NS	52.0 (37.0–77.0)	NS	68.0 (53.0–72)
Social functioning	87.5 (75.0–100)	**0.0007**	50.0 (40.6–87.5)	**0.0009**	100 (65.6–100)
Pain	80.0 (67.5–100)	NS	65.0 (45.0–100)	NS	90.0 (57.5–100)
General health	55.0 (35.0–70.0)	**0.0443**	42.5 (20.0–58.8)	NS	45.0 (36.3–63.8)
Health change	50.0 (50.0–75.0)	NS	50.0 (50.0–75.0)	NS	50.0 (50.0–75.0)

Values in bold mean that they are statistically significant (*p* < 0.05).

NS, nonsignificant.

Pain, general health, and health change was impaired in all the 3 groups but without any between-group differences (Fig. 2).

#### Correlation Between ENT Scores and Disease Activity

**FIGURE 3:**
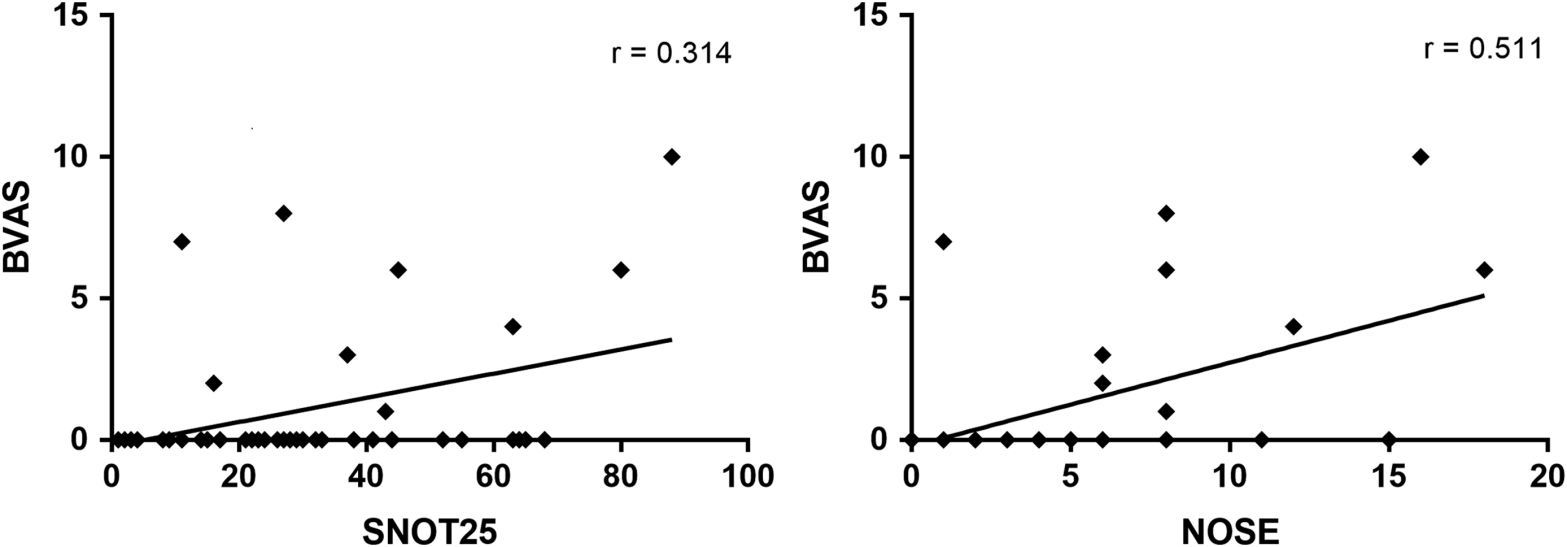
Correlations between BVAS and SNOT-25 (left), and BVAS and NOSE (right) in AAV patients' cohort.

Focusing on disease activity, there was a significant correlation between BVAS and SNOT-25 (*p* = 0.037; *r* = 0.314). The correlation resulted even stronger with the 3 ENT-specific items of the SNOT-25 (*p* = 0.006; *r* = 0.405), whereas no correlation came to light between BVAS and SNOT-22. Similarly, a positive correlation between BVAS and NOSE (*p* = 0.004; *r* = 0.511) was also observed (Fig. 3). No further correlations were evident between ENT scores and age, disease duration, or damage accrual.

#### Influence of the Therapy

In the ENT-AAV group, there were no differences in SNOT-22, SNOT-25, and SF-36 scores between users and nonusers of topical douches or topical nasal steroids. No differences were detected in all the scores considering current immunosuppressive treatment or glucocorticoids.

## DISCUSSION

In the last decade, AAV has become a chronic condition,^8,9^ and many patients experience persistent disease activity or a relapsing disease together with long-term exposure to therapies leading to irreversible damage.^12^ Such clinical course significantly impact on the general QoL of patients who are prone to develop depression, anxiety,^17^ and work disability.^20^

Primary outcomes are usually rated using the physician-based BVAS score,^31^ which assesses disease activity, even if AAV patients often report different perceptions of the disease. The Outcome Measures in Rheumatology core set of outcome measurements for use in clinical trials in AAV included the generic SF-36 patient-reported outcome to evaluate QoL.^36^ However, generic patient-reported outcome can lack specificity,^37^ especially regarding the features of vasculitis and its specific organ involvement; thus, focused questionnaires have been used in the present study.^26,33,34^

Ear, nose, and throat and renal involvement are prominent organ conditions found in 50% to 80% of patients with AAV and reflect the granulomatous and vasculitic components, respectively.^7^ However, recent observations indicate a clinical interaction between ENT and renal disease. When compared with patients with no ENT involvement, those with ENT involvement showed less frequent renal disease, better renal function,^2,23^ and a younger age at diagnosis.^2^ This is consistent with our results.

Chronic rhinosinusitis has been previously demonstrated to have a significant impact on the QoL, comparable to other common chronic diseases such as rheumatoid arthritis, chronic obstructive pulmonary disease, and insulin-dependent diabetes.^38^

In the present study, the burden of sinonasal morbidity (assessed with SNOT score) in AAV patients with ENT involvement is at least as significant as that of CRS, confirming the results of Srouji and colleagues.^26,39^ This is also important considering that AAV-related sinonasal morbidity is regarded by many physicians to be of less impact in the setting of multisystem disease. The same results were observed when the NOSE score was applied.

Among the reported nasal symptoms in the sinonasal AAV group, “hearing loss,” “loss of olfactory/taste sensation,” “tiredness/fatigue,” “feeling frustrated/fatigued/irritable,” and “feeling sad” were commonly rated as one of the most troublesome symptoms from a list of 25, confirming the relevance of such symptoms in this condition. Moreover, considering SNOT in its 4 domains (rhinological, facial/audiological, sleep/wake rhythm, and emotional/physical disorders), the physical and emotional disorders domain was significantly impaired in AAV patients compared with those with CRS, confirming a poorer psychological-emotional well-being in vasculitic subjects.

Most importantly, we found that patients with AAV who specifically have active sinonasal disease report poorer general QoL scores (SF-36) compared with the unaffected counterparts. This is consistent with the results of Srouji et al,^26^ who reported an impairment especially of social functioning, perhaps as a result of the stigma of constant purulent rhinorrhea, embarrassing epistaxis, or nasal deformity from cartilage destruction. Similar conclusions were obtained with asthenia, limitations in daily/work activities because of emotional state (such as feeling depressed and anxious), and physical health. Interestingly, the psychological-emotional domain was statistically comparable between patients with ENT and non-ENT involvement, but significantly poorer when compared with the CRS group, suggesting a general impairment in AAV patients, possibly disease or treatment related.

We also found a positive correlation between both SNOT and NOSE scores and disease activity, measured by means of BVAS version 3. This result is consistent with the findings of Piccirillo et al,^33^ who reported that SNOT score has a moderate responsiveness to the modification of disease activity in patients with CRS.

The high impact of ENT symptoms on QoL detected in this study confirms the importance of their early treatment through specific local approach. However, even if similar conclusions have been already reported more than 15 years ago,^26^ to date no randomized, prospective studies evaluating the efficacy of local treatments are available. Interestingly, no significant differences in QoL were noted when considering systemic therapy, including both glucocorticoids and immunosuppressants. It has to be pointed out that, in our cohort, at the time of assessment, only one AAV patient was currently treated with rituximab, which is nowadays recommended for induction and maintenance of remission in relapsing and severe AAV.

Although dealing with a rare autoimmune disease, the limitations of the present study include the relatively small number of patients and the lack of information on their work/employment condition. In addition, the study design does not allow performing a risk factor analysis, and prospective, longitudinal studies are warranted. However, the present findings confirm the results of previous studies, and for the first time, patient-reported outcomes were assessed through multiple specific questionnaires. Further studies are warranted to confirm results on larger cohorts and on other conditions as eosinophilic GPA.

## CONCLUSIONS

The QoL in patients with AAV is significantly reduced, especially in the presence of ENT involvement. The AAV-related nasal morbidity is consistent and comparable to the high levels reported by patients with CRS. This involvement seems to affect more the social functioning, leading to limitation in daily/work activities. Although the role of systemic therapy in inducing and maintaining clinical remission has been largely confirmed in the literature, the efficacy of topical therapy and the influence of such treatments on the QoL of patients remains still unclear.
